# A Versatile Anti-CXCR5 Monoclonal Antibody (Cx_5_Mab-6) for Basic Research and Diagnosis

**DOI:** 10.3390/cimb48090901

**Published:** 2026-09-03

**Authors:** Airi Nomura, Hiroyuki Suzuki, Saori Okuno, Haruto Yamamoto, Reina Ito, Yukari Ogura, Kai Shimizu, Takuro Nakamura, Miyuki Yanaka, Saori Handa, Mika K. Kaneko, Yukinari Kato

**Affiliations:** Department of Antibody Drug Development, Tohoku University Graduate School of Medicine, 2-1 Seiryo-machi, Aoba-ku, Sendai 980-8575, Miyagi, Japan; nomura.airi.p3@dc.tohoku.ac.jp (A.N.); saori.okuno.a8@tohoku.ac.jp (S.O.); haruto.yamamoto.b8@tohoku.ac.jp (H.Y.); ito.reina.r7@dc.tohoku.ac.jp (R.I.); ogura.yukari.t4@dc.tohoku.ac.jp (Y.O.); shimizu.kai.p1@dc.tohoku.ac.jp (K.S.); takuro.nakamura.a2@tohoku.ac.jp (T.N.); miyuki.yanaka.c5@tohoku.ac.jp (M.Y.); saori.handa.d3@tohoku.ac.jp (S.H.); mika.kaneko.d4@tohoku.ac.jp (M.K.K.)

**Keywords:** monoclonal antibody, CXCR5, Cell-Based Immunization and Screening, flow cytometry, Western blotting, immunohistochemistry

## Abstract

The G protein-coupled receptor, CXC chemokine receptor 5 (CXCR5), is predominantly expressed on B cells located in the secondary lymphoid tissues, follicular helper T cells, and lymphoma cells. Binding to its ligand, CXCL13, mediates cell migration and regulates lymphocyte trafficking. Aberrant CXCL13/CXCR5 expression and signaling have been implicated in tumor progression, autoimmune diseases, and chronic inflammatory disorders. Therefore, specific mAbs against CXCR5 are expected to be useful for diagnosis and therapeutic applications. In this study, novel anti-human CXCR5 mAbs (Cx_5_Mabs) were developed through flow cytometry-based high-throughput screening. One clone, Cx_5_Mab-6 (IgG_2b_, κ), recognized CXCR5-overexpressed Chinese hamster ovary (CHO)-K1 cells but did not react with the other five CXCR receptors-overexpressed CHO-K1 cells in flow cytometry. Additionally, Cx_5_Mab-6 recognized endogenous CXCR5 in the human Burkitt lymphoma Raji cell line. The dissociation constant (*K*_D_) values of Cx_5_Mab-6 for CHO/CXCR5 and Raji were 3.4 × 10^−9^ M and 1.2 × 10^−10^ M, respectively. Furthermore, Cx_5_Mab-6 is useful for Western blotting and can detect CXCR5 in human lymphoma tissue by immunohistochemistry. These findings suggest that Cx_5_Mab-6 is versatile for basic research and has potential applications in clinical diagnosis.

## 1. Introduction

Chemokines are classified into four subfamilies (CC, CXC, CX3C, and XC) based on the number and arrangement of conserved N-terminal cysteine residues [1,2]. Chemokine receptors are seven-transmembrane G protein-coupled receptors (GPCRs) that initiate intracellular signaling upon ligand binding [1,3,4]. The chemokine-receptor system plays essential roles in lymphocyte proliferation, differentiation, organization, and immune responses [5,6].

CXCR5, formerly known as Burkitt lymphoma receptor 1, is predominantly expressed on normal B cells in secondary lymphoid tissues, including the spleen, lymph nodes, tonsil, and Peyer’s patches [7]. CXCR5 is also expressed on follicular helper T (Tfh) cells and lymphoma cells [8]. Upon binding to its ligand, CXCL13, CXCR5 mediates cell migration and plays a critical role in lymphocyte trafficking [9,10]. CXCL13 is produced by multiple cell types, including follicular dendritic cells, Tfh cells, macrophages, and epithelial cells [11]. In secondary lymphoid tissues, CXCL13 orchestrates lymphoid architecture by recruiting B cells into follicles. During immune responses, CXCL13 guides Tfh and B cells to the light zone of germinal centers (GCs), where Tfh cells help B cells undergo affinity maturation and differentiate into long-lived plasma cells or memory B cells [11].

The CXCL13-CXCR5 axis has been implicated in the pathogenesis of several autoimmune diseases, including rheumatoid arthritis, systemic lupus erythematosus (SLE), multiple sclerosis, Sjögren’s disease (SjD), and inflammatory bowel disease (IBD) [12,13]. In autoimmune diseases, aberrant CXCL13 expression induces ectopic lymphoid proliferation and promotes the production of pathogenic autoantibodies [14]. In rheumatoid arthritis, CXCL13 enhances endothelial progenitor cell recruitment and vascular endothelial growth factor expression, thereby promoting synovial angiogenesis [15]. In lupus nephritis, CXCL13 stimulates mesangial cell proliferation and proinflammatory mediator secretion in podocytes [11]. In patients with IBD, serum CXCL13 levels are significantly elevated compared with those in healthy controls [16]. In a dextran sodium sulfate-induced mouse model of colitis, CXCL13 expression was markedly increased in the colon. Genetic deficiency of CXCL13 suppressed colitis development by reducing the migration of CD4^+^ CXCR5^+^ T cells to the mesenteric lymph nodes and promoting the accumulation of regulatory B cells in the colon [16]. These results support the CXCL13-CXCR5 axis as a potential therapeutic target for IBD.

Aberrant CXCL13/CXCR5 expression and signaling have been implicated in tumor progression [17]. CXCR5 is expressed in several B-cell lymphomas and in a subset of T-cell lymphomas, generally reflecting the tumor’s normal cellular origin [18]. CXCR5 is also expressed in solid tumors, including lung cancer [19], breast cancer [20], colorectal cancer [21], and prostate cancer [22]. The CXCL13-CXCR5 axis promotes tumor proliferation and invasion by activating the phosphatidylinositol 3-kinase, Raf/MEK/ERK, focal adhesion kinase/paxillin, and DOCK2/Rac/JNK pathways [23]. These findings suggest that CXCR5 is a promising therapeutic target, and that an antibody capable of selectively eliminating CXCR5-positive malignant tumors may be an effective treatment strategy [17]. Anti-CXCR5 chimeric antigen receptor (CAR) T therapies targeting B- and T-cell lymphomas have been developed in preclinical studies [24,25].

The Cell-Based Immunization and Screening (CBIS) method is a high-throughput flow cytometry-based approach for generating monoclonal antibodies (mAbs). Using the CBIS method, we generated several anti-chemokine receptor mAbs suitable for flow cytometry (https://www.med-tohoku-antibody.com/topics/antibody_bank.htm, accessed on 28 August 2026). Most mAbs that recognize conformational epitopes are not suitable for Western blotting and immunohistochemistry (IHC). A few mAbs, such as the anti-mouse CCR1 mAb (C_1_Mab-6), can be used in Western blotting [26]. In this study, we employed the CBIS method to develop a highly versatile anti-CXCR5 mAb for basic research including flow cytometry, Western blotting, and IHC.

## 2. Materials and Methods

### 2.1. Cell Lines

Chinese hamster ovary (CHO)-K1, human glioblastoma (GBM) LN229, and mouse myeloma P3X63Ag8U.1 (P3U1) cell lines were obtained from the American Type Culture Collection (ATCC, Manassas, VA, USA). Human Burkitt lymphoma Raji cell line was obtained from the RIKEN BioResource Research Center (Ibaraki, Japan). The cell lines were maintained as described previously [27]. P3U1, Raji, and CHO-K1 were cultured in Roswell Park Memorial Institute-1640 medium (Nacalai Tesque, Inc., Kyoto, Japan), supplied with 100 U/mL penicillin, 100 μg/mL streptomycin, 0.25 μg/mL amphotericin B (Nacalai Tesque, Inc., Kyoto, Japan), and 10% heat-inactivated fetal bovine serum (FBS; Thermo Fisher Scientific, Inc., Waltham, MA, USA). LN229 were cultured in Dulbecco’s Modified Eagle Medium (Nacalai Tesque, Inc.), supplied with the above supplements. All the cells were cultured in a humidified incubator at 37 °C with 5% CO_2_.

### 2.2. Plasmid Construction and Establishment of Stable Transfectants

The cDNAs of human *CXCR1* (NM_000634.3), *CXCR2* (NM_001557.4), *CXCR5* (NM_001716.5) were synthesized by Eurofins Genomics KK (Tokyo, Japan). Genes encoding human *CXCR3* (NM_001504.2), *CXCR4* (NM_003467.3), and *CXCR6* (NM_006564.2) were obtained from the RIKEN BioResource Research Center. The cDNAs were subcloned into the pCAG-Ble vector. These plasmids were transfected into CHO-K1 or LN229 cells, and stable transfectants were sorted using anti-CXCR1 mAb (clone 8F1/CXCR1, BioLegend, San Diego, CA, USA), anti-CXCR2 mAb (clone 5E8/CXCR2, BioLegend), anti-CXCR3 mAb (clone G025H7, BioLegend), anti-CXCR4 mAb (clone 2B11/CXCR4, BD Biosciences, Franklin Lakes, NJ, USA), anti-CXCR5 mAb (J252D4, BioLegend), and anti-CXCR6 mAb (clone K041E5, BioLegend) using the Neon transfection system (Thermo Fisher Scientific, Inc.). Finally, CXCR1-overexpressed CHO-K1 (CHO/CXCR1), CXCR2-overexpressed CHO-K1 (CHO/CXCR2), CXCR3-overexpressed CHO-K1 (CHO/CXCR3), CXCR4-overexpressed CHO-K1 (CHO/CXCR4), CXCR5-overexpressed CHO-K1 (CHO/CXCR5), CXCR6-overexpressed CHO-K1 (CHO/CXCR6), and CXCR5-overexpressed LN229 (LN229/CXCR5) were established. The stable transfectants were maintained as described previously [27].

### 2.3. Production of Hybridomas

All animal experiments were performed in accordance with relevant institutional guidelines and regulations, with appropriate measures taken to minimize pain, distress, and suffering. The experimental protocol was approved by the Animal Care and Use Committee of Tohoku University (Permit No. 2019NiA-001). Mice were housed under specific pathogen-free conditions with an 11 h light/13 h dark cycle and had free access to food and water throughout the study. Their general health and well-being were assessed daily. A loss of more than 25% of the initial body weight was defined as a humane endpoint. At the end of the experiment, mice were euthanized by cervical dislocation, and death was verified by the absence of respiration and cardiac activity.

CHO/CXCR5 was used as an immunogen. Female BALB/cAJcl mice (CLEA Japan, Tokyo, Japan) were intraperitoneally immunized with CHO/CXCR5 (1 × 10^8^ cells/injection) mixed with 2% Alhydrogel adjuvant (InvivoGen, San Diego, CA, USA). Following three additional weekly immunizations (1.0 × 10^8^ cells/injection), a booster dose (1 × 10^8^ cells/injection) was administered two days before spleen excision. Hybridomas were produced as previously described [28]. A mAb, Cx_5_Mab-6, was purified from hybridoma culture supernatant maintained in serum-free Hybridoma-SFM medium (Thermo Fisher Scientific, Inc.) using Ab-Catcher Extra affinity resin (ProteNova, Kagawa, Japan).

### 2.4. Flow Cytometric Analysis

CHO-K1, CHO/CXCR1, CHO/CXCR2, CHO/CXCR3, CHO/CXCR4, CHO/CXCR5, CHO/CXCR6, LN229, LN229/CXCR5, and Raji were harvested with 1 mM EDTA and washed with blocking buffer [0.1% bovine serum albumin in phosphate-buffered saline (PBS)]. The cells were incubated with primary mAbs for 30 min at 4 °C. The cells were then stained with Alexa Fluor 488-conjugated anti-mouse IgG (1:2000; Cell Signaling Technology, Inc., Danvers, MA, USA). Isotype control mouse IgG_1_ and IgG_2b_ mAbs (CvMab-62 and RdMab-20, respectively) were described previously [29,30]. Flow cytometric data were acquired on an SA3800 Cell Analyzer (Sony Corp., Tokyo, Japan). Cells were gated on forward scatter (FSC) and side scatter (SSC), and fluorescence intensity was analyzed using FlowJo software (Ver. 10.8.1, BD Biosciences, San Jose, CA, USA).

### 2.5. Determination of Dissociation Constant Values Using Flow Cytometry

To evaluate the binding affinity of Cx_5_Mab-6 and J252D4, CHO/CXCR5 and Raji were incubated with serially diluted mAbs, followed by staining with Alexa Fluor 488-conjugated anti-mouse IgG (1:200 dilution). Fluorescence signals were acquired by flow cytometry, and the geometric mean fluorescence intensity (GeoMean) was analyzed using FlowJo software. The dissociation constant (*K*_D_) was estimated by fitting the binding curves (antibody concentration versus GeoMean) to a one-site binding model implemented in GraphPad Prism 6 (GraphPad Software, Inc., La Jolla, CA, USA).

### 2.6. Western Blotting

CHO-K1 and CHO/CXCR5 cell lysates were prepared and were boiled in sodium dodecyl sulfate sample buffer containing 2-mercaptoethanol (Nacalai Tesque, Inc.). Western blotting was performed as described previously [29]. Cx_5_Mab-6 (1 μg/mL), J252D4 (1 μg/mL), and an anti-isocitrate dehydrogenase 1 (IDH1) mAb (clone RcMab-1-mG_1_, 1 μg/mL, for internal control) were used as primary mAbs. Horseradish peroxidase-conjugated anti-mouse IgG (1:1000; Agilent Technologies Inc., Santa Clara, CA, USA) was used as the secondary mAb. Chemiluminescence signals were developed using Pierce™ ECL Plus (Thermo Fisher Scientific, Inc.) or ImmunoStar LD (FUJIFILM Wako Pure Chemical Corporation, Osaka, Japan). The signals were imaged using ChemiDoc Touch MP (Bio-Rad Laboratories, Inc., Berkeley, CA, USA).

### 2.7. Immunohistochemistry Using Cell Blocks and Tissue Arrays

All IHC procedures were performed on the VENTANA BenchMark ULTRA PLUS (Roche Diagnostics, Indianapolis, IN, USA). Formalin-fixed, paraffin-embedded (FFPE) CHO-K1 and CHO/CXCR5 sections were prepared as described previously [27]. A human lymphoma tissue microarray (LM241b) was purchased from US Biomax Inc. (Rockville, MD, USA). Sections were stained with 0.5 μg/mL of Cx_5_Mab-6, RdMab-20, or J252D4 using the BenchMark ULTRA PLUS with the ultraView Universal DAB Detection Kit (Roche Diagnostics, Indianapolis, IN, USA). IHC staining intensity was independently assessed by two investigators in a blinded manner.

## 3. Results

### 3.1. Development of Anti-CXCR5 mAbs by the CBIS Method

**Figure 1:**
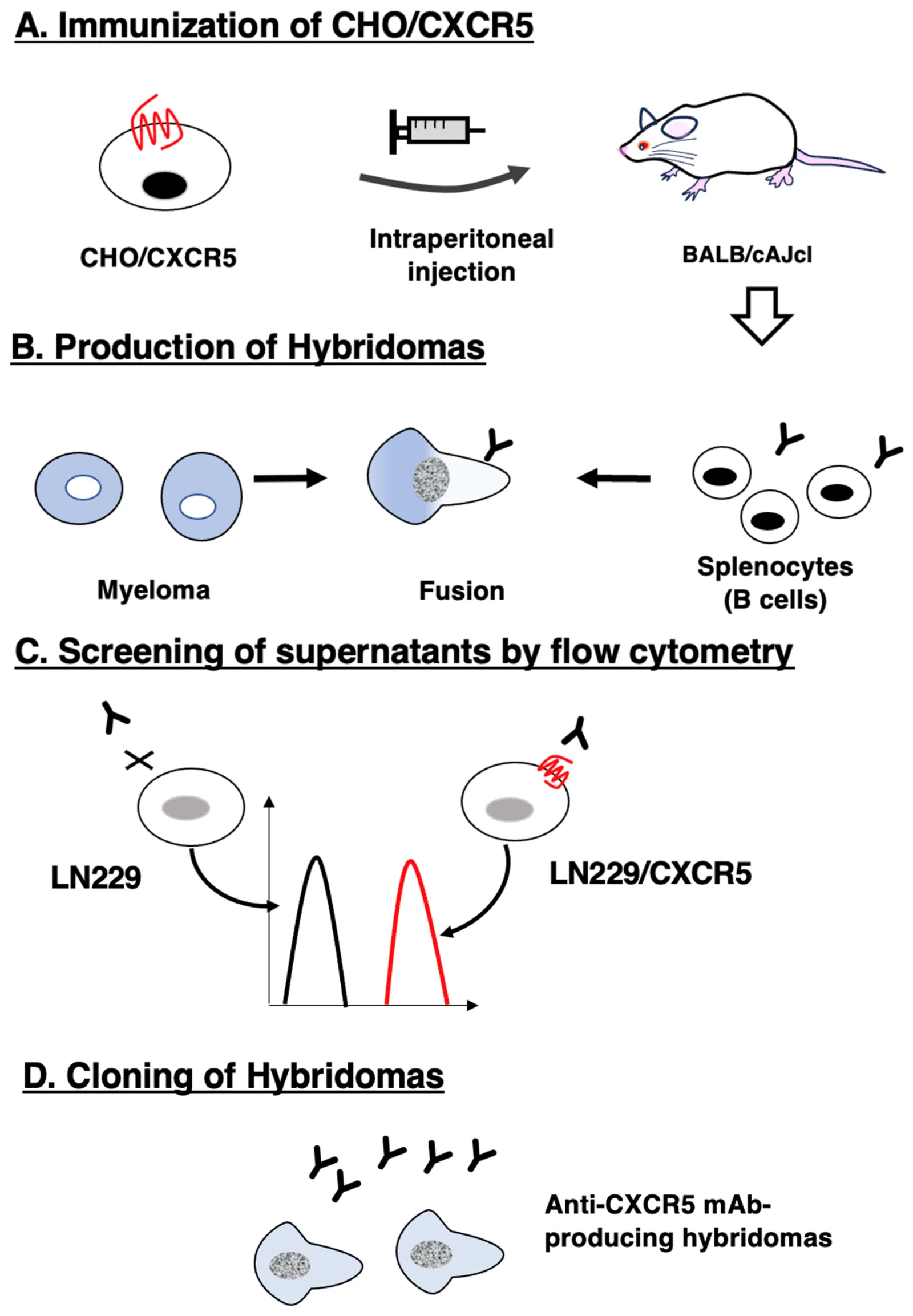
Schematic representation of anti-CXCR5 mAb production. (**A**) CHO/CXCR5 was injected into BALB/cAJcl mice intraperitoneally. (**B**) After five immunizations, spleen cells were fused with P3U1. (**C**) The supernatants from hybridomas were screened by flow cytometry using LN229/CXCR5 and LN229 cells. (**D**) An anti-CXCR5 mAb-producing hybridoma clone (Cx_5_Mab-6) was established through limiting dilution.

As described in Section 2.2, CHO/CXCR5 was prepared as an immunogen. CHO/CXCR5 (1 × 10^8^ cells/mouse) was immunized five times into two BALB/cAJcl mice (Figure 1A). Subsequently, hybridomas were generated by fusing the splenocytes with myeloma P3U1 (Figure 1B). The supernatants of hybridoma were screened to identify those positive for LN229/CXCR5 and negative for parental LN229 (Figure 1C). As a result, 13 positive wells out of 958 (1.4%) were identified. Limiting dilution was performed to clone hybridomas producing anti-CXCR5 mAb (Figure 1D). Finally, three clones (Cx_5_Mab-5, Cx_5_Mab-6, and Cx_5_Mab-7) were successfully obtained. After validation of reactivity and specificity by flow cytometry and other methods, such as Western blotting and immunohistochemistry, clone Cx_5_Mab-6 (IgG_2b_, κ) was selected and purified as described in Section 2.3.

### 3.2. Flow Cytometry Analysis of Cx_5_Mab-6 and J252D4 Against CXCR5-Overexpressed Cells

**Figure 2:**
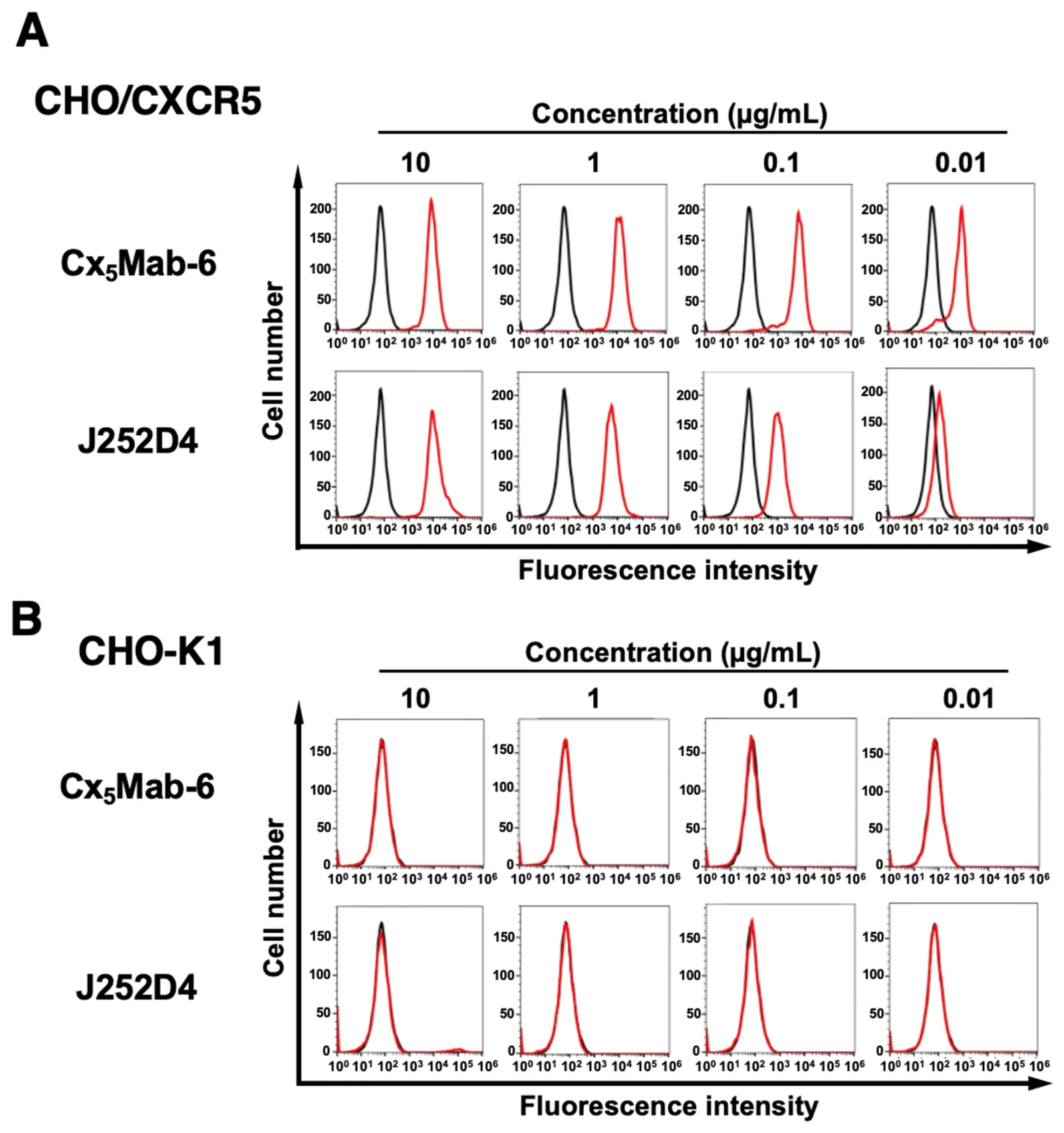
Flow cytometric analysis using Cx_5_Mab-6 and J252D4. CHO/CXCR5 (**A**) and CHO-K1 (**B**) were treated with Cx_5_Mab-6 and J252D4 at the indicated concentrations (red) or with blocking buffer (black, negative control). The mAbs-treated cells were incubated with Alexa Fluor 488-conjugated anti-mouse IgG. Fluorescence data were collected using the SA3800 Cell Analyzer. The experiments were conducted twice.

We next performed flow cytometry using Cx_5_Mab-6 against CHO/CXCR5, CHO-K1, LN229/CXCR5, and LN229. Cx_5_Mab-6 reacted with CHO/CXCR5 in a dose-dependent manner from 10 to 0.01 μg/mL (Figure 2A). In contrast, Cx_5_Mab-6 did not recognize CHO-K1 even at 10 μg/mL (Figure 2B). A commercially available anti-CXCR5 mAb (clone J252D4) also recognized CHO/CXCR5 but not CHO-K1 (Figure 2A,B). Furthermore, Cx_5_Mab-6 and J252D4 reacted with LN229/CXCR5 in a dose-dependent manner (Supplementary Figure S1A). In contrast, Cx_5_Mab-6 and J252D4 did not recognize LN229 (Supplementary Figure S1B).

### 3.3. The Specificity of Cx_5_Mab-6 Using CXCRs-Overexpressed CHO-K1

**Figure 3:**
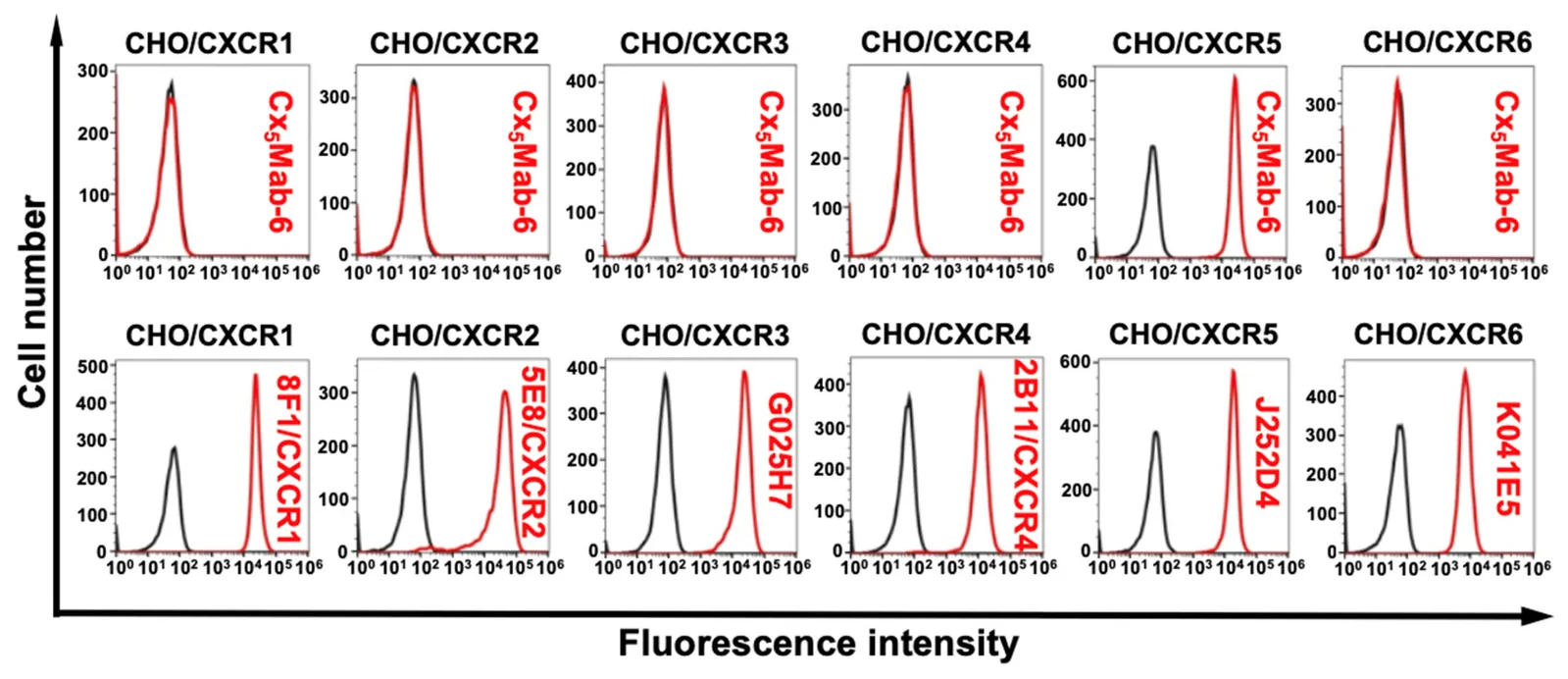
Flow cytometry analysis of Cx_5_Mab-6 in CXCRs-overexpressed CHO-K1. (Upper panels) The CXCR1, CXCR2, CXCR3, CXCR4, CXCR5, and CXCR6-overexpressed CHO-K1 were treated with 1 µg/mL of Cx_5_Mab-6 (red) or with control blocking buffer (black, negative control), followed by treatment with anti-mouse IgG conjugated with Alexa Fluor 488. (Lower panels) Each CXCR expression was confirmed by 1 µg/mL of an anti-CXCR1 mAb (clone 8F1/CXCR1), an anti-CXCR2 mAb (clone 5E8/CXCR2), an anti-CXCR3 mAb (clone G025H7), an anti-CXCR4 mAb (clone 2B11/CXCR4), an anti-CXCR5 mAb (clone J252D4), and an anti-CXCR6 mAb (clone K041E5), followed by the treatment with Alexa Fluor 488-conjugated secondary mAbs. The fluorescence data were collected using the SA3800 Cell Analyzer. The experiments were conducted twice.

To investigate the specificity of Cx_5_Mab-6 among CXCR members, CHO-K1 cell lines which overexpressed other CXCRs, including CXCR1, CXCR2, CXCR3, CXCR4, or CXCR6, were additionally established. As shown in Figure 3, Cx_5_Mab-6 reacted with CHO/CXCR5 but did not react with other CXCRs overexpressed in CHO-K1. The cell surface expression of CXCRs was confirmed by each mAb. These results indicate that Cx_5_Mab-6 is a specific mAb to CXCR5 among those CXCRs.

### 3.4. Flow Cytometry Analysis of Cx_5_Mab-6 and J252D4 Against an Endogenous CXCR5-Expressing Cell Line

**Figure 4:**
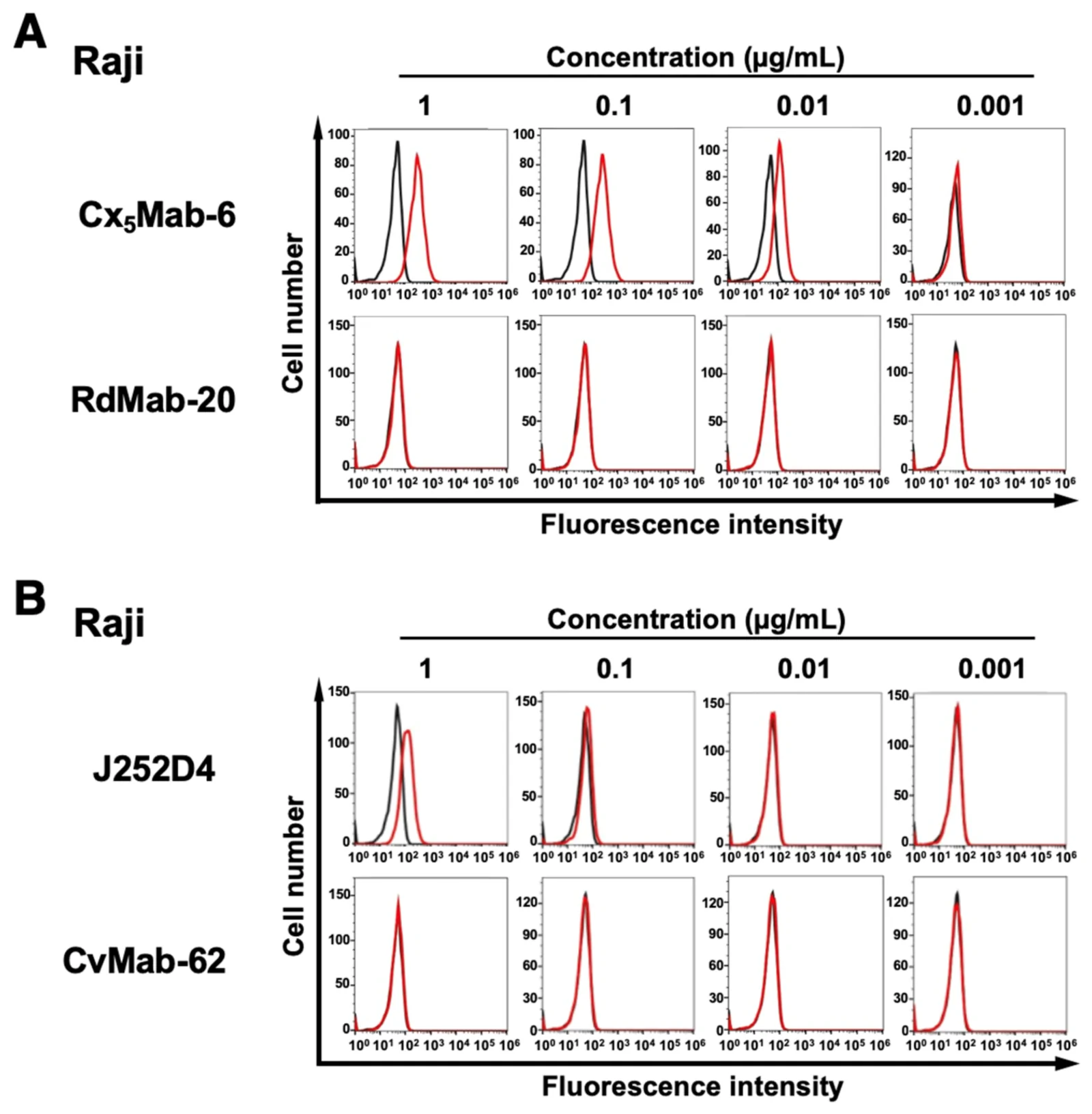
Flow cytometric analysis using Cx_5_Mab-6 and J252D4 against an endogenous CXCR5-positive cell line. (**A**) Raji was treated with Cx_5_Mab-6, IgG_2b_ isotype control (RdMab-20) at the indicated concentrations (red), or blocking buffer (black, negative control). (**B**) Raji was treated with J252D4, IgG_1_ isotype control (CvMab-62) at the indicated concentrations (red), or blocking buffer (black, negative control). The mAb-treated cells were incubated with Alexa Fluor 488-conjugated anti-mouse IgG. Fluorescence data were collected using the SA3800 Cell Analyzer. The experiments were conducted twice.

The endogenous CXCR5 in a human Burkitt lymphoma Raji cell line was investigated using Cx_5_Mab-6 and J252D4. Cx_5_Mab-6 reacted with Raji in a dose-dependent manner from 1 to 0.01 μg/mL. In contrast, an IgG_2b_ isotype control mAb (RdMab-20) did not recognize Raji even at 1 μg/mL (Figure 4A). Clone J252D4 also recognized Raji at 1 μg/mL but an IgG_1_ isotype control mAb (CvMab-62) did not (Figure 4B). These results indicate that Cx_5_Mab-6 could detect endogenous CXCR5 in Raji cell line.

### 3.5. Determination of Binding Affinity of Cx_5_Mab-6 to CXCR5-Positive Cells

**Figure 5:**
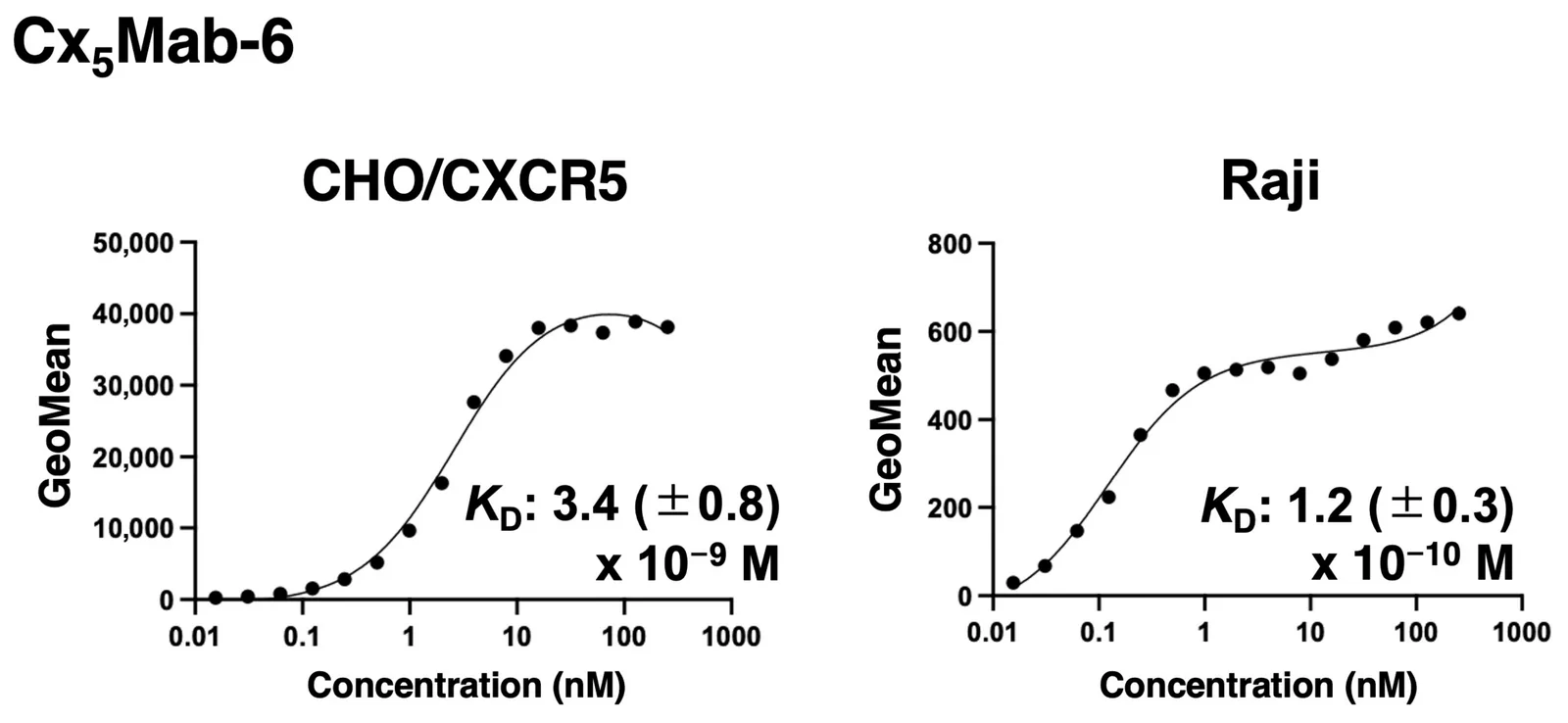
Determination of the binding affinity of Cx_5_Mab-6 by flow cytometry. CHO/CXCR5 and Raji cells were incubated with serially diluted Cx_5_Mab-6, then reacted with Alexa Fluor 488-conjugated anti-mouse IgG. Geometric mean fluorescence values were measured using the SA3800 Cell Analyzer and FlowJo software. Average *K*_D_ values (± standard deviation) from three independent measurements were calculated using GraphPad PRISM 6. Representative graphs are shown.

The binding affinity of Cx_5_Mab-6 was measured with CHO/CXCR5 and Raji as described in Section 2.5. The *K*_D_ values for Cx_5_Mab-6 with CHO/CXCR5 and Raji were 3.4 (±0.8) × 10^−9^ M and 1.2 (±0.3) × 10^−10^ M, respectively (Figure 5). J252D4 showed lower binding affinity compared to Cx_5_Mab-6 [1.6 (±0.3) × 10^−8^ M for CHO/CXCR5 and 1.1 (±0.1) × 10^−8^ M for Raji, Supplementary Figure S2]. These results indicated that Cx_5_Mab-6 has high binding affinity for exogenous and endogenous CXCR5.

### 3.6. Western Blotting Using Cx_5_Mab-6 and J252D4

**Figure 6:**
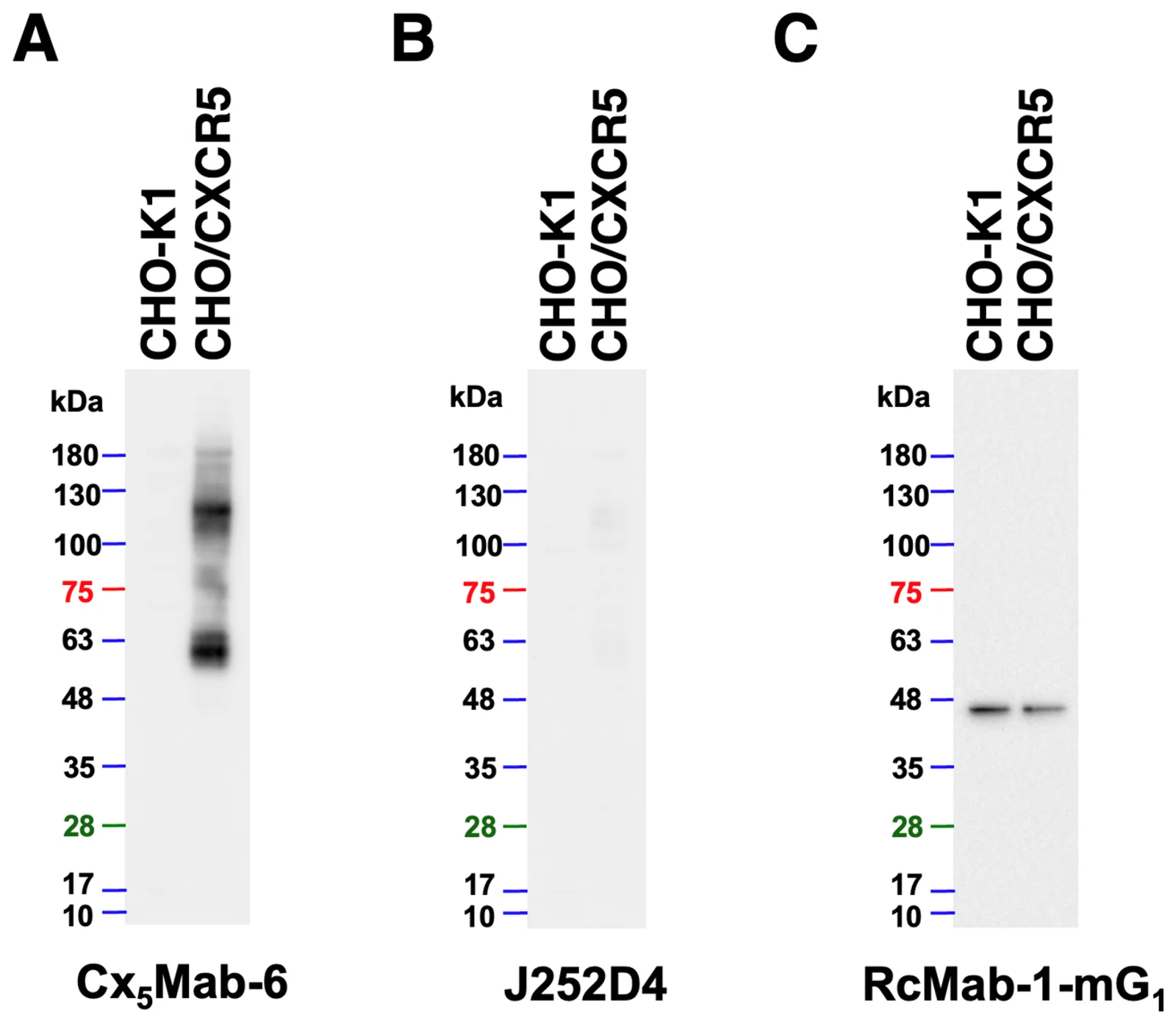
Western blotting using Cx_5_Mab-6 and J252D4. Cell lysates (10 μg/lane) from CHO-K1 and CHO/CXCR5 were electrophoresed and transferred to polyvinylidene difluoride membranes. The membranes were incubated with 1 μg/mL of Cx_5_Mab-6 (**A**), 1 μg/mL of J252D4 (**B**), or 2 μg/mL of RcMab-1-mG_1_ (an anti-IDH1 mAb) (**C**), followed by the treatment with anti-mouse IgG conjugated with horseradish peroxidase. The exposure condition in (**A**,**B**) was same. The experiments were conducted twice.

Western blotting was performed using Cx_5_Mab-6 and J252D4. Whole-cell lysates from CHO-K1 and CHO/CXCR5 were analyzed. Cx_5_Mab-6 mainly detected 60 and 120 kDa bands in CHO/CXCR5, but not in CHO-K1 (Figure 6A). Although J252D4 showed a similar band pattern, the intensity was weak compared to that of Cx_5_Mab-6 (Figure 6B). Figure 6C shows an internal control, isocitrate dehydrogenase 1 (IDH1), detected by RcMab-1-mG_1_. These results demonstrate that Cx_5_Mab-6 can detect CXCR5 with high sensitivity in Western blotting.

### 3.7. Immunohistochemistry Using Cx_5_Mab-6 and J252D4 in Formalin-Fixed Paraffin-Embedded Cell Blocks

**Figure 7:**
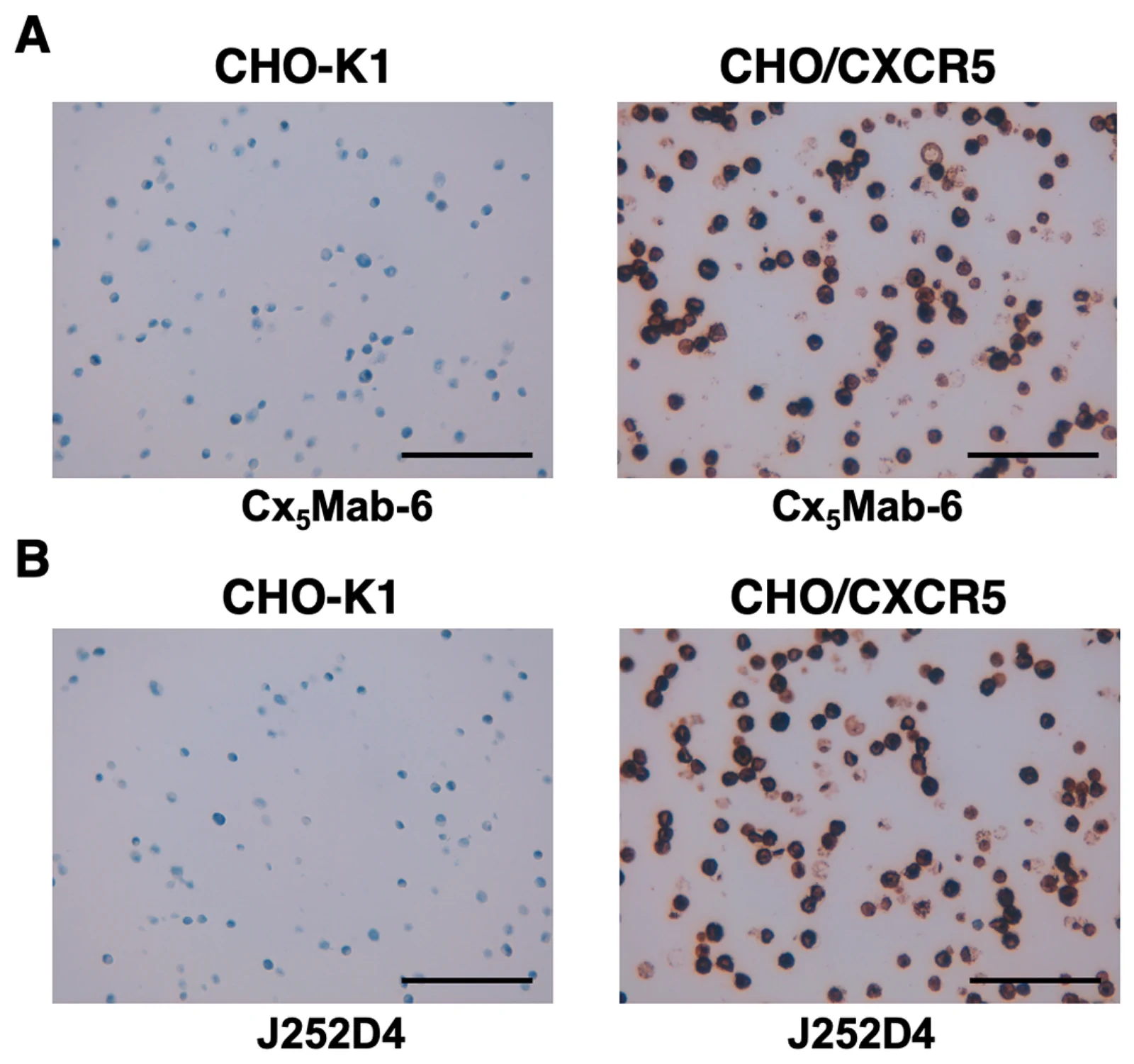
Immunohistochemistry using Cx_5_Mab-6 and J252D4 in formalin-fixed paraffin-embedded cell blocks. CHO/CXCR5 and CHO-K1 sections were treated with 0.5 μg/mL of Cx_5_Mab-6 (**A**) or J252D4 (**B**). The staining was performed using BenchMark ULTRA PLUS with the ultraView Universal DAB Detection Kit, Scale bar = 100 μm. The experiments were conducted twice.

We assessed whether Cx_5_Mab-6 and J252D4 are suitable for IHC of FFPE sections from CHO-K1 and CHO/CXCR5. Cx_5_Mab-6 and J252D4 showed strong membranous and cytoplasmic staining in CHO/CXCR5 but not in CHO-K1 (Figure 7A,B). These results suggest that Cx_5_Mab-6 can detect CXCR5 in IHC of FFPE sections of cultured cells.

### 3.8. Immunohistochemistry Using Cx_5_Mab-6 in Formalin-Fixed Paraffin-Embedded Tumor Tissue

**Figure 8:**
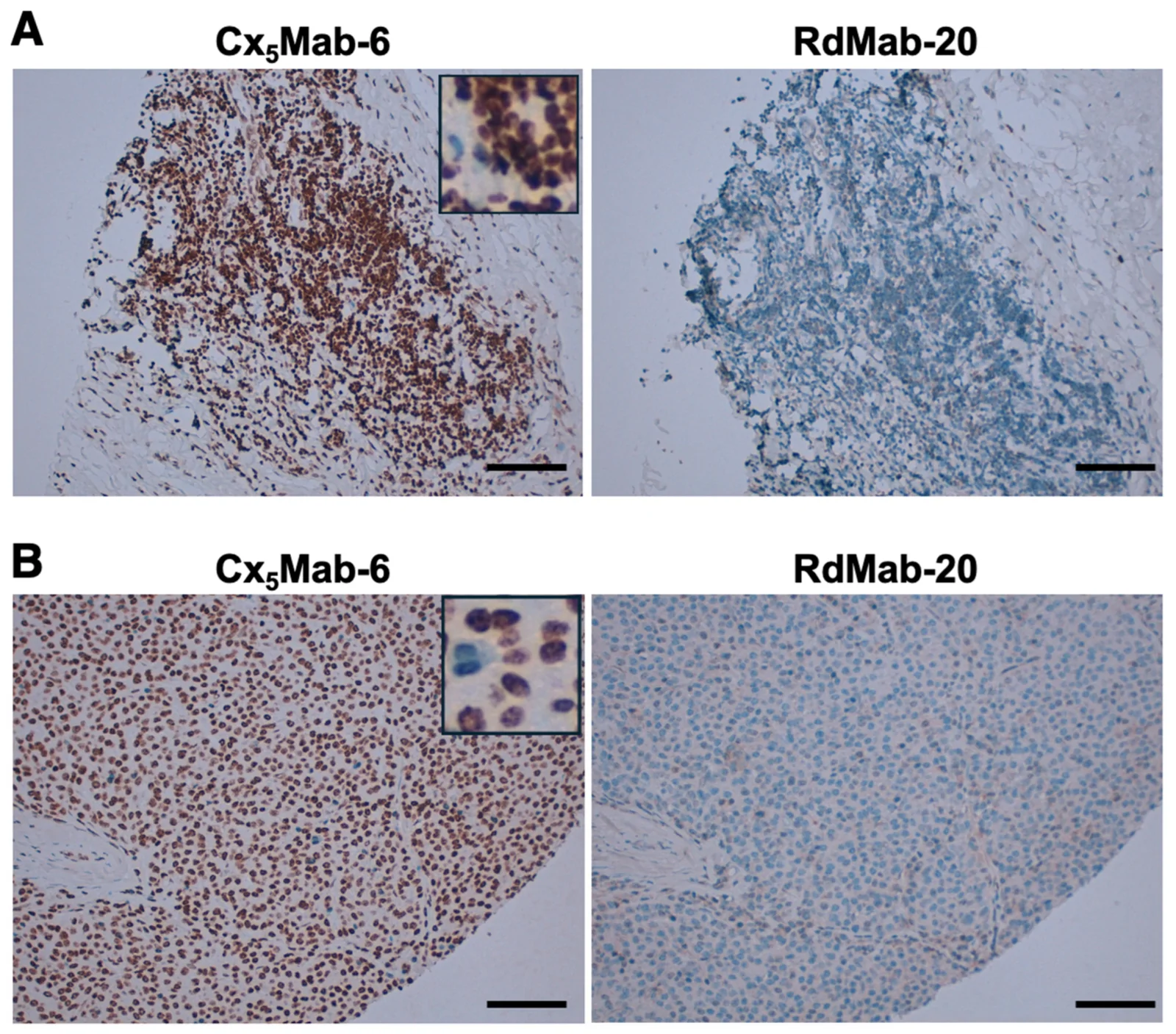
Immunohistochemistry using Cx_5_Mab-6 in a lymphoma tissue array. Sequential sections of human lymphoma tissue array (LM241b) were treated with 0.5 μg/mL of Cx_5_Mab-6 or isotype control IgG_2b_ (RdMab-20). Normal tonsil (**A**) and plasmacytic lymphoma (**B**) were shown. The high-magnification images were inserted at the top right corners of each image. The staining was performed using BenchMark ULTRA PLUS with the ultraView Universal DAB Detection Kit, Scale bar = 100 μm.

CXCR5 is predominantly expressed on tonsillar B cells and lymphoma cells. Using a lymphoma tissue array, IHC was next performed. Cx_5_Mab-6 showed positive staining in lymphoid follicle of tonsil (Figure 8A) and a case of plasmacytic lymphoma (Figure 8B), but the isotype control mAb did not. The high-magnification images inserted at the top right corners indicated the presence of CXCR5-positive and negative cells. Similar staining was observed in diffuse large-B-cell lymphoma and diffuse T-cell lymphoma (Supplementary Figure S3). Table 1 summarizes the result. This result indicates that Cx_5_Mab-6 is suitable to detect endogenous CXCR5 in FFPE lymphoma tissues by IHC.

## 4. Discussion

This study demonstrated a novel anti-CXCR5 mAb, Cx_5_Mab-6, established by the CBIS method (Figure 1). Cx_5_Mab-6 recognized both exogenous and endogenous CXCR5 in flow cytometry (Figure 2 and Figure 4) without cross-reactivity to other CXCRs (Figure 3), indicating that Cx_5_Mab-6 is useful for the specific detection and isolation of the CXCR5-positive cells by fluorescence-activated cell sorting. CXCR5 typically positions CD4+ Tfh cells within the B-cell zone to aid in antibody production [31]. Secondary lymphoid organs also harbor a functionally distinct subset of memory CD8+ T cells defined by the expression of CXCR5 [32]. A landmark study identified CXCR5+ CD8+ T cells in human tonsils and showed that they promoted IgG production by B cells in vitro, indicating a direct B-cell helper function [33]. Furthermore, the CXCR5+ CD8+ T cells are emerging as important mediators of antiviral immunity during chronic infection [34]. CXCR5+ CD8+ T cells enriched in human tonsils exhibited tissue-resident characteristics and express Granzyme K [35]. CXCR5 expression is especially enriched in tonsillar CD8+ T cells specific for latent Epstein–Barr virus (EBV) antigens and is associated with a programmed death 1 (PD-1) + stem-like tissue-resident phenotype, suggesting that this subset contributes to local immune surveillance against EBV [35]. Cx_5_Mab-6 is suitable for IHC in cell blocks (Figure 7) and human tonsil (Figure 8) using an automated slide-staining system, VENTANA BenchMark ULTRA PLUS. Through a multiple staining technique, Cx_5_Mab-6 would contribute to the identification of CXCR5+ CD4+ T cells or CXCR5+ CD8+ T cells in IHC.

The tumor immune contexture reflects the density, composition, functional status, and spatial organization of tumor-infiltrating leukocytes and can serve as a source of information for assessing prognosis and predicting therapeutic response to immunotherapy [36]. Immunohistochemical studies have demonstrated the favorable clinical implications of the presence of CD8+ T cells in many solid tumors [36]. The tumor-infiltrating CD8+ T cells are largely dysfunctional [37], and further single-cell sequencing of the whole transcriptome analysis identified CXCR5+ CD8+ T cells, which possess gene networks responsible for stem-like plasticity and cytotoxicity with a partial exhausted phenotype [38]. The presence of CXCR5+ CD8+ T cells is associated with better overall survival in patients with lung cancer [38], pancreatic cancer [39], gastric cancer [40], hepatocellular carcinoma [41], and colorectal cancer [42]. Furthermore, the presence of CXCR5+ CD8+ T cells indicates high responsiveness to immune checkpoint blockade therapy in some tumors [43,44,45]. Therefore, Cx_5_Mab-6 is thought to be useful for predicting responsiveness to immune checkpoint blockade therapy.

SjD and SLE exhibit several common serological characteristics indicative of systemic autoimmune disease, including dysregulated B-cell responses, the generation of autoantibodies such as antinuclear antibodies and anti-Ro/SSA antibodies, a pronounced interferon-related gene signature, and persistent production of proinflammatory cytokines in circulation [46,47,48]. Beyond these systemic abnormalities, SjD is characterized by tissue- and organ-specific infiltration of lymphocytes, local production of proinflammatory cytokines, and impairment of glandular function [49,50]. SjD and SLE have been implicated to share genetic risk at 11q23.3 (DDX6-CXCR5 locus) [51]. The functional single-nucleotide polymorphism (SNP) analyses revealed that the SNPs showed cell type-specific and allele-specific effects on nuclear protein binding and enhancer/promoter activity in immune and salivary gland/kidney epithelial cells. These results suggest that the SNPs at the DDX6-CXCR5 locus influence the common mechanisms of autoimmunity, including interferon signaling (DDX6) and lymphocytic infiltration of disease-target tissues (CXCR5) in SjD and SLE [52]. Cx_5_Mab-6 would be useful for diagnosis to detect the aberrant B-cell activation and ectopic lymphoid organization in SjD and SLE.

As shown in the result of Western blotting, Cx_5_Mab-6 mainly detected two major bands (60 and 120 kDa) in the reduced condition of CHO/CXCR5 lysate (Figure 6A). As shown in a previous study, in vitro translated unmodified CXCR5 in rabbit reticulocytes revealed 38 kDa protein, and an approximately 60 kDa CXCR5 band was detected in CXCR5-overexpressed 293 cell lysate [53]. Growing evidence has demonstrated that GPCRs are modified by glycosylation [54]. Treatment of tunicamycin, an inhibitor of *N*-glycan synthesis, reduced the molecular weight of CXCR5 [55]. These results suggest that *N*-glycosylation of CXCR5 is involved in the formation of a 120 kDa band in CHO/CXCR5. Additionally, a more than 120 kDa band was mainly detected by Cx_5_Mab-6 in Raji cell lysate. Further generation of CXCR5-knockout Raji is essential to clarify the endogenous CXCR5 expression and the status of CXCR5 glycosylation in Raji cells.

Identification of mAb epitope is essential to understand the property of mAb. We previously reported the flow cytometry-based epitope mapping using extracellular domain-substituted mutants and point mutants. Using the strategy, we identified an epitope in the extracellular loop 2 in an anti-mouse CCR1 mAb (clone S15040E) [56] and an epitope in the N-terminal region in another anti-mouse CCR1 mAb (clone C_1_Mab-6) [57]. The strategy could contribute to the identification of Cx_5_Mab-6 epitope, which may help the understanding of the structure or biological property of Cx_5_Mab-6 in the future.

## 5. Conclusions

Cx_5_Mab-6 was developed using the CBIS method and can be used in flow cytometry, Western blotting, and IHC. Therefore, Cx_5_Mab-6 is useful for basic research and diagnosis. Although specificity among CXCR members has been confirmed, cross-reactivity with other GPCRs has not been investigated. Additionally, the epitope and biological activity of Cx_5_Mab-6 have not been determined. cDNA cloning of Cx_5_Mab-6 is ongoing and will contribute to the generation of recombinant Cx_5_Mab-6 or to the development of a CAR for tumor therapy.

## Figures and Tables

**Table 1:** Immunohistochemistry of a lymphoma tissue array (LM241b) by Cx_5_Mab-6.

No.	Age	Sex	Pathology Diagnosis	Organ	Cx_5_Mab-6
1	71	M	Diffuse B-cell lymphoma of neck	LN	1+
2	63	M	Plasmacytic lymphoma of left armpit	LN	3+
3	50	M	Diffuse B-cell lymphoma	Colon	3+
4	64	F	Diffuse large-B-cell lymphoma of left groin	LN	3+
5	43	F	Diffuse large-B-cell lymphoma of left groin	LN	3+
6	16	M	Mucosa-associated B-cell lymphoma	Colon	1+
7	58	M	Mantle cell lymphoma of neck	LN	1+
8	64	M	Lymphoid plasma cell lymphoma of neck	LN	2+
9	51	M	Diffuse large-B-cell lymphoma of right armpit	LN	2+
10	28	M	Diffuse T-cell lymphoma of left neck	LN	3+
11	45	M	Normal tonsil tissue	Tonsil	3+

1+, weak intensity; 2+, moderate intensity; 3+, strong intensity. LN, lymph node.

## Data Availability

The raw data supporting the conclusions of this article will be made available by the authors on request.

## Supplementary Materials

The following supporting information can be downloaded at https://www.mdpi.com/article/10.3390/cimb48090901/s1.
