# Supplementary material for: A Versatile Anti-CXCR5 Monoclonal Antibody (Cx5Mab-6) for Basic Research and Diagnosis
Source: Curr Issues Mol Biol. 2026 Sep 3;48(9):901. doi: 10.3390/cimb48090901 (PMC13605946; doi:10.3390/cimb48090901)
Supplement: Supplementary file 1 [file cimb-48-00901-s001.zip › cimb-4524569-supplementary.pdf]

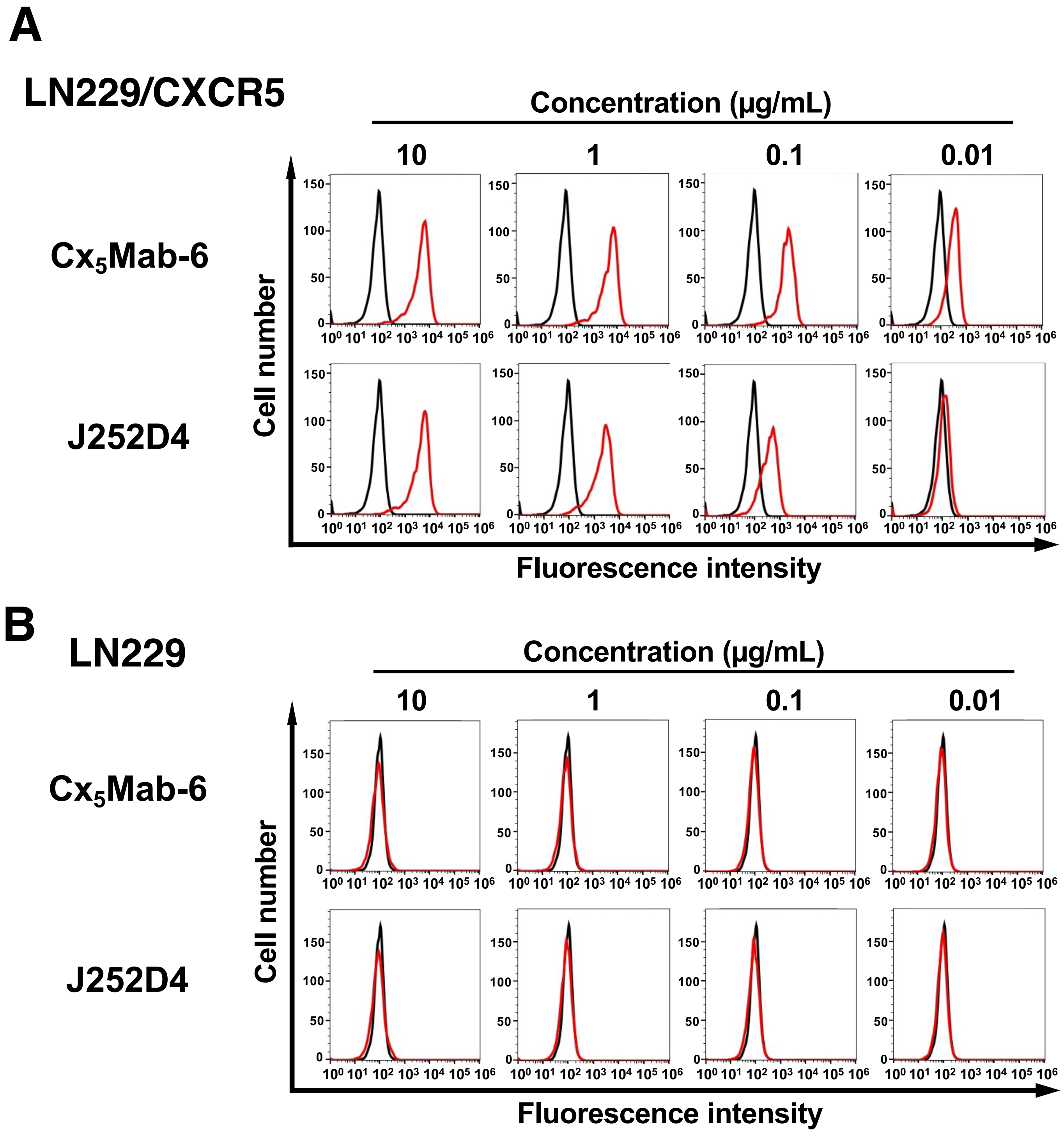

Supplementary Figure S1. Flow cytometric analysis using Cx$_5$Mab-6 and J252D4 in LN229/CXCR5 and LN229. LN229/CXCR5 (A) and LN229 (B) were treated with Cx$_5$Mab-6 and J252D4 at the indicated concentrations (red) or with blocking buffer (black, negative control). The mAbs-treated cells were incubated with Alexa Fluor 488-conjugated anti-mouse IgG. Fluorescence data were collected using the SA3800 Cell Analyzer. The experiments were conducted twice.

J252D4

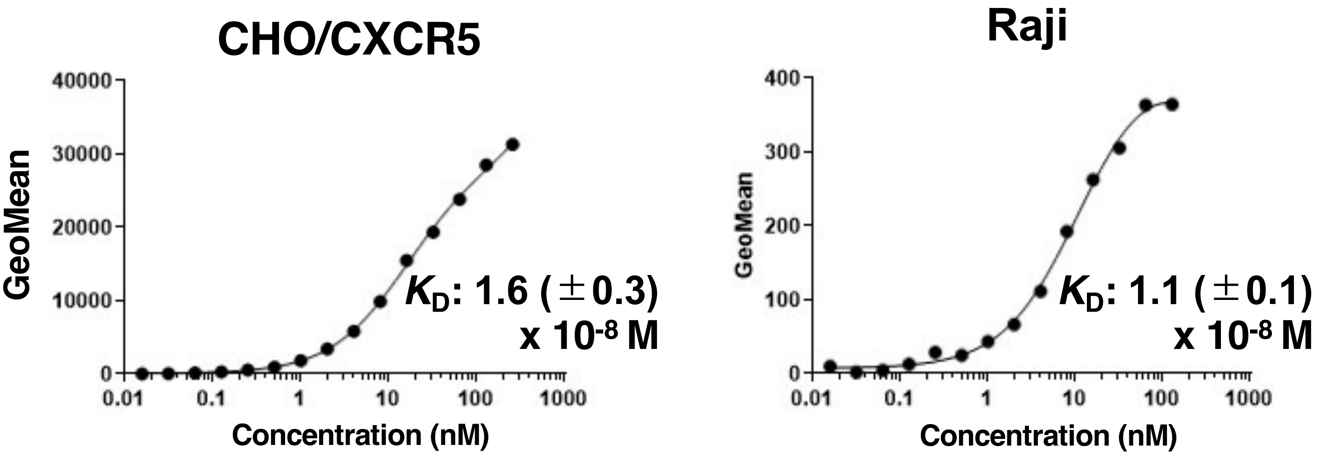

Supplementary Figure S2. Determination of the binding affinity of J252D4 by flow cytometry. CHO/CXCR5 and Raji cells were incubated with serially diluted J252D4, then reacted with Alexa Fluor 488-conjugated anti-mouse IgG. Geometric mean fluorescence values were measured using the SA3800 Cell Analyzer and FlowJo software. Average K_D values (± standard deviation) from three independent measurements were calculated using GraphPad PRISM 6. Representative graphs are shown.

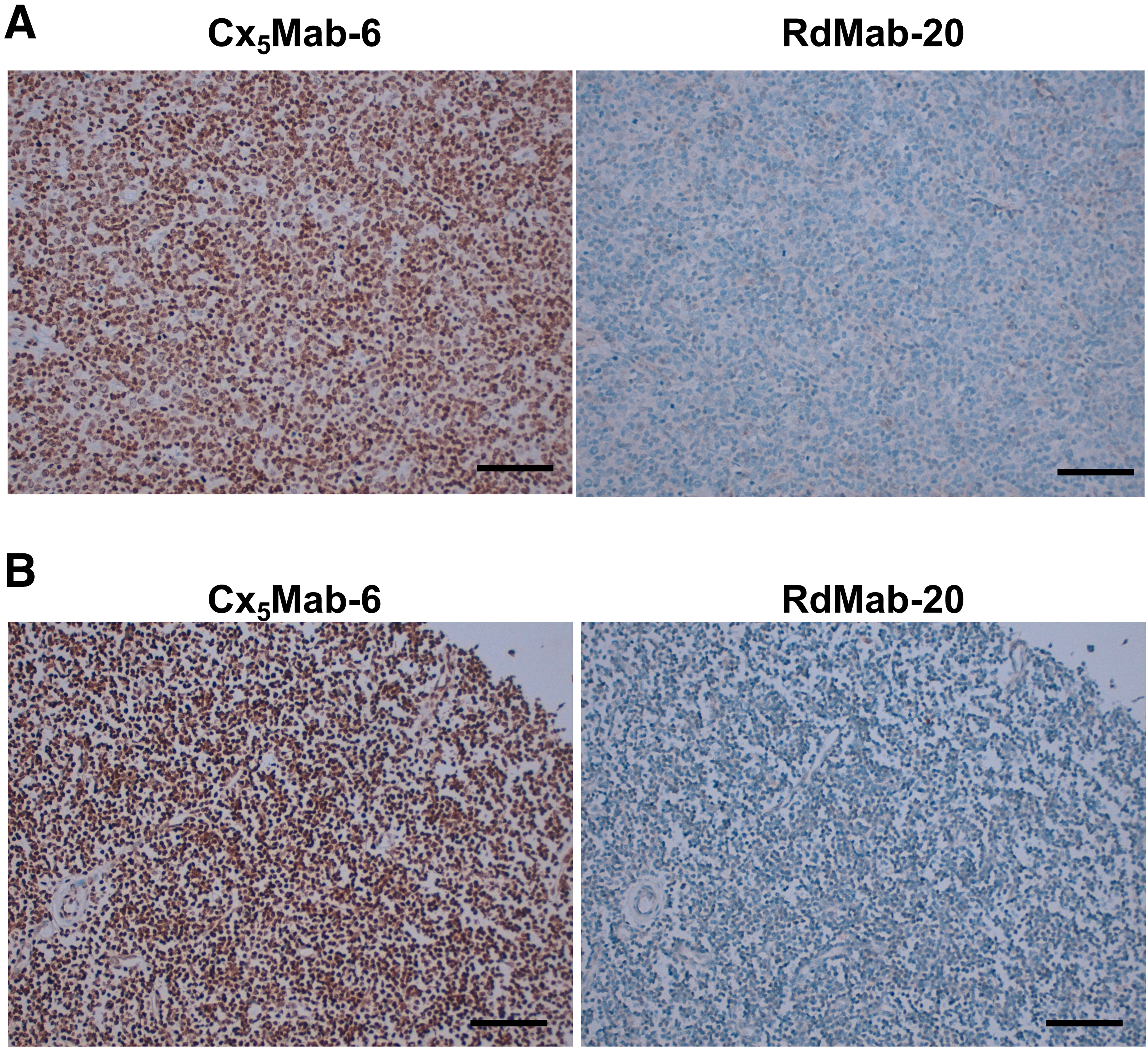

Supplementary Figure S3. Immunohistochemistry using Cx_5Mab-6 in a lymphoma tissue array. Sequential sections of human a lymphoma tissue array (LM241b) were treated with 0.5 µg/mL of Cx_5Mab-6 or 0.5 µg/mL of isotype control IgG_{2b} (RdMab-20). Diffuse large-B cell lymphoma (A) and diffuse T cell lymphoma (B) were shown. The staining was performed using BenchMark ULTRA PLUS with the ultraView Universal DAB Detection Kit, Scale bar = 100 µm.
